# Spatio-temporal predictions of COVID-19 test positivity in Uppsala County, Sweden: a comparative approach

**DOI:** 10.1038/s41598-022-19155-y

**Published:** 2022-09-07

**Authors:** Vera van Zoest, Georgios Varotsis, Uwe Menzel, Anders Wigren, Beatrice Kennedy, Mats Martinell, Tove Fall

**Affiliations:** 1 Department of Information Technology, Uppsala University, P.O. Box 337, 751 05 Uppsala, Sweden; 2 Department of Medical Sciences, Molecular Epidemiology and Science for Life Laboratory, Uppsala University, 751 85 Uppsala, Sweden; 3 Department of Public Health and Caring Sciences, Uppsala University, 751 22 Uppsala, Sweden

**Keywords:** Infectious diseases, Statistics

## Abstract

Previous spatio-temporal COVID-19 prediction models have focused on the prediction of subsequent number of cases, and have shown varying accuracy and lack of high geographical resolution. We aimed to predict trends in COVID-19 test positivity, an important marker for planning local testing capacity and accessibility. We included a full year of information (June 29, 2020–July 4, 2021) with both direct and indirect indicators of transmission, e.g. mobility data, number of calls to the national healthcare advice line and vaccination coverage from Uppsala County, Sweden, as potential predictors. We developed four models for a 1-week-window, based on gradient boosting (GB), random forest (RF), autoregressive integrated moving average (ARIMA) and integrated nested laplace approximations (INLA). Three of the models (GB, RF and INLA) outperformed the naïve baseline model after data from a full pandemic wave became available and demonstrated moderate accuracy. An ensemble model of these three models slightly improved the average root mean square error to 0.039 compared to 0.040 for GB, RF and INLA, 0.055 for ARIMA and 0.046 for the naïve model. Our findings indicate that the collection of a wide variety of data can contribute to spatio-temporal predictions of COVID-19 test positivity.

## Introduction

Τhe spread of COVID-19 often manifests itself in the form of geographical cluster outbreaks^1–3^. This pattern is likely due to transmission taking place in interior public spaces such as public recreational facilities, public transport vehicles, religious venues and shopping centers^3^ or other buildings where social interactions occur^1–4^. In order to curb local outbreaks and stop any continuous transmission, local health authorities need to react to local needs and be flexible to adapt COVID-19 testing, contact-tracing of the chain of transmission, and communication campaigns accordingly. However, to quickly identify the areas in need of such targeted actions, better tools are needed, such as precise epidemic geospatial predictions to locate local outbreaks.

Several research teams have focused on strictly data-driven approaches to make forecasts regarding the spread of COVID-19, by searching for temporal patterns on the most recent available data using statistical methods and extrapolating past trajectories into the future^5–7^. The predictions resulting from a time-series analysis usually yield a wide predictive range of accuracy, but they are particularly useful for short-term forecasting when the prediction window is between 1 and 10 days^5–7^. However, the majority of studies dealing with COVID-19 predictions lack high geographical resolution and focus on forecasting confirmed cases^6–10^ with some also investigating fatalities^8,11^. Nevertheless, a finer geographical resolution is essential to guide local testing strategies while the test positivity has been suggested by the World Health Organization (WHO) as one of the main criteria that need to be considered in the assessment of epidemic control^12^. For example, a high test positivity can be an indication of increased community transmission and delayed case identification.

Meanwhile, an alternative to single modelling is the so-called ensemble forecasting method which has been utilized with varying success in the modelling of several infectious diseases^13^. It combines two or more distinct prediction models by incorporating their outputs into one using weighted averages^5,11^ and it has the potential to give better predictive power and accuracy compared to the performance of each model studied separately^14^.

The aim of this study is to develop and evaluate the performance of four different statistical models and one ensemble model in predicting COVID-19 test positivity among areas with small population sizes ranging from 992 to 19,226 inhabitants within Uppsala County in Sweden from July 2020 to June 2021 using a 1-week prediction window. The models that were considered to cover a range of statistical methods included gradient boosting (GB), random forest (RF), autoregressive integrated moving average (ARIMA) and integrated nested laplace approximations (INLA). Finally, the distinct results from all four models were merged into a weighted ensemble to investigate whether a combination would improve the predictive performance. Models varied depending on the covariates and the methods used for parameter estimation, and the spatial and/or temporal autocorrelation structure included in the model.

## Methods

### Dataset

We studied data originating from the Uppsala County in Sweden which is the fifth most populated county (388,394 inhabitants) in Sweden with a total land surface area of 818,962 hectares and a population density of 47.7 inhabitants/km^2^^15^. The county encompasses eight municipalities which were further divided into 50 service point areas. A service point area was defined as a group of postal codes that are served by the same parcel collection and drop-off service as indicated by the national postal service PostNord. In Sweden, such service points are often located in the same building as major grocery and retail stores. In this way, the service point areas resemble boundaries within which people connect and meet.

All the data used in the prediction models was collected by the Uppsala County Council and the CRUSH Covid initiative—a multidisciplinary team of researchers from Uppsala University—with the aim to assist the local health authorities to curb local outbreaks of COVID-19 by monitoring temporal disease trends over key spatial areas. By using a diversity of data sources, a comprehensive database has consistently been curated by CRUSH Covid every Monday and descriptive statistics on each data source are communicated to health officials and the public every week.

We considered the following types of data to build our prediction models: (1) the geographical location of the service point areas; (2) the demographic characteristics of each area such as population size, gender distribution and neighborhood deprivation index (NDI)^16^; (3) weekly direct indicators of the spread of COVID-19 based on the number and the results of COVID-19 RT-PCR tests performed in each area, the adjacent areas, as well as on a national level; (4) weekly hospitalization and ICU beds occupied by patients in each area as well as on a national level; (5) weekly indirect indicators such as the number of calls to the national 1177 Healthcare Advice Line and the 112 emergency number assessed as suspected COVID-19^17^; (6) information on vaccination coverage (cumulative proportion of residents that had received 1 or 2 doses of a COVID-19 vaccine approved by the European Medicines Agency (EMA) at least 3 or 2 weeks prior, respectively); and (7) indicators of increased contacts within a social context in workplaces, retail areas and recreational spaces, as well as changes in time spent at home using Google Mobility data on a municipality level. A complete overview of all variables is included in Table S1 in the Supplementary Materials.

The time-variable data was collected consistently on a weekly basis for all variables between June 29, 2020 and July 4, 2021 with the exception of the number of COVID-19-related calls to the national 1177 Healthcare Advice Line for which data was available starting from November 16, 2020.

### Variable of interest

The outcome was set as the prediction of test positivity (${y}_{i,t}$), that is proportion of confirmed COVID-19 positive cases, out of all COVID-19 tests conducted during the following week (*t*) for each of the service-point neighborhoods (*i*). The seven-day prediction window was chosen for three main reasons: (1) most of the data was only available once a week and aggregated on a weekly level and, (2) the public health agency of the Uppsala County Council would have one bi-weekly meeting and wished to know where they needed to increase testing efforts in the next week and thus daily estimates were less relevant and (3) a shorter-term forecast tends to be more precise than longer ones^11^. In the “Prediction models” section, we describe the four prediction models used in this study, based on GB, RF, ARIMA and INLA.

### Performance evaluation

To evaluate the different models, we considered a moving time series window of training and validation data as new data comes in throughout the pandemic. We started with an initial training dataset of 20 weeks and use this to predict ${y}_{i,t}$ for week $t=21$ and all areas $i$. We computed ${RMSE}_{t}$ as the Root Mean Squared Error for week $t$ over all areas $i$ to compare performance of the different models:

1 $${RMSE}_{t}= \sqrt{\frac{{\sum }_{i=1}^{{N}_{i}}{({\widehat{y}}_{i,t}-{y}_{i,t})}^{2}}{{N}_{i}}},$$

where ${\widehat{y}}_{i,t}$ is the predicted positivity and ${y}_{i,t}$ is the observed positivity in area $i$ and week $t$, and ${N}_{i}$ is the total number of areas. As new data comes in every week, all previous data is used for training and the ${RMSE}_{t}$ is updated using validation data of week $t$. Thus, for each model we created a time series of ${RMSE}_{t}$ over week $t$ in $(21\dots {N}_{t})$. A lower ${RMSE}_{t}$ value indicates better performance.

Finally, we considered a naïve model to evaluate the performance of all models compared to a baseline model. In the naïve model, the predictions for area $i$ and week $t$ are simply based on the values observed in area $i$ in the previous week $t-1$:

2 $${\widehat{y}}_{i,t}={y}_{i,t-1}.$$

Similar to the other models, we evaluate ${RMSE}_{t}$ over week $t$ in $(21\dots {N}_{t})$. To compare the significance of the difference between the models, the entire RMSE time series of the different models were compared to each other using a two-tailed paired Wilcoxon signed-rank test with significance level $\alpha =0.05$. To test whether the models improved performance compared to the baseline (naïve) model, the entire RMSE time series of the different models were compared to the RMSE time series of the naïve model using a one-tailed paired Wilcoxon signed-rank test with significance level $\alpha =0.05$.

### Prediction models

We compared the results of four prediction models. Considering that there is no known reference standard for predicting COVID-19 test-positivity, we selected these models in an attempt to cover a range of statistical methods that would include the use of external regressors, time series analysis as well as spatio-temporal analysis. More specifically, gradient boosting and random forest are selected as tree-based models, allowing for a large number of external regressors while able to automatically detect feature importance. An ARIMA model is selected for time-series analysis without external regressors, as this model is commonly used in epidemiological literature on time series forecasting^18^. The INLA model has been selected for analysis of the spatio-temporal covariance structure in the data.

#### Multivariate regression using gradient boosting

For training of the GB model, 23 predictor variables of the data set described in Table S1 (Supplementary Materials) were considered. We used the “gbm” package in R, which implements the approach described by Friedman^19,20^. The GB machine uses short decision trees (“decision stumps”) as the underlying mechanism for variable selection and regression. We tuned four parameters linked to GB in order to ensure that optimum model fits were achieved, namely: (1) the number of trees used for each model fit (“*n.trees*”), (2) the maximum depth of each tree (“*interaction.depth*”), (3) the minimum number of observations in the terminal nodes (“*n.minobsinnode*”), and (4) the shrinkage parameter applied to each tree (“*shrinkage*”). Detailed information regarding these parameters can be found in Greenwell et al.^21^. We made sure that the search space was large enough by comparing the resulting optimum values with the search ranges chosen. This was done in an iterative manner. On the one hand, our goal was to enclose the optimum values completely within the search space. On the other hand, in order to speed up calculations, the search space was narrowed down as much as possible around the calculated optimum values.

#### Multivariate regression using random forest

For training of the RF model, 23 predictor variables of the data set described in Table S1 (Supplementary Materials) were considered, similar to the GB model. The RF regression model^22^ was implemented using the package “randomforest”^23^ in R. The RF learner uses ensembles of decision trees in order to train models and to make predictions. We calculated 1000 decision trees (*ntree* = 1000) for each RF model. The number of variables used in each split of the decision trees (*mtry*) was tuned at each stage of the cumulative calculation, because the optimum values varied between 1 and 6, depending on the number of weeks included in the training set. It was ensured that the maximum value of the tuning range for *mtry* was bigger than all computed optimum values, in order to make sure that the search space was big enough. The minimum value of the tuning range for *mtry* was set to one in each step of the cumulative procedure. During training, parameter tuning was carried out for each time point based on repeated tenfold cross validation over the spatial domain, i.e. iteratively dividing the service point areas into 90% for training and 10% for testing until all service areas have been used for testing after 10 iterations. The parameter set providing the smallest RMSE was considered to represent the best model.

#### Univariate time series predictions using ARIMA

The ARIMA approach considers a pure time series model, based on the temporal autocorrelation in test positivity, without the use of other potential predictors. After mean centering, we consider the following equations. First, we consider the autoregressive part (AR):

3 $${\widehat{y}}_{i,t}={\sum }_{\tau =1}^{T}{\varphi }_{\tau }{y}_{i,t-\tau },$$

for time lag $\tau$ in $\left(1\dots T\right)$, where $T$ is the total number of previously observed time points (weeks) used in the model, i.e. the order of the AR part. In Eq. (3), ${\widehat{y}}_{i,t}$ is the predicted positivity for week $t$ and area $i$ and $\varphi$ is a scaling coefficient. Next, we consider the Moving Average (MA) part. In the MA part, the error terms in the AR equation are estimated according to the following equation:

4 $${\widehat{y}}_{i,t}={\mu }_{i}+{\sum }_{q=1}^{Q}{\theta }_{q}{\varepsilon }_{i,t-q},$$

for time lag $q$ in $(1\dots Q)$, where $Q$ is the order of the moving average model. Furthermore, ${\theta }_{q}$ is defined as the model parameter for time lag $q$ and ${\mu }_{i}$ represents the drift. If ${\theta }_{q}$ is significantly different from zero ($\alpha =0.05$), the estimated value is added to the predicted positivity ${\widehat{y}}_{i,t}$ from Eq. (3). The two equations described above are called ARMA. For ARMA to provide robust estimates, the time series is required to be stationary, which means that the input has a constant mean and a constant variance across the entire time series. For non-stationary time series, differentiation is made to a level where the time series becomes stationary in differentiated form. The ARIMA model refers to an ARMA analysis performed on differentiated time series data.

Each ARIMA ($p, d, q$) model is defined by three parameters $p$, $d$ and $q$, where $p$ is the number of parameters for timelags, $d$ number of differentiations, and $q$ is the number of MA parameters. One example of a simple non-stationary model is ARIMA (0, 1, 0): a random walk model, i.e. a first-order autoregressive model:

5 $${\widehat{y}}_{i,t}={\mu }_{i}+{y}_{i,t-1},$$

where ${\mu }_{i}$ reflects the long-term drift.

Another example is the ARIMA (1, 1, 0), which includes a first-order non-seasonal component and is suitable when there is autocorrelation of the residuals from the random walk model:

6 $${\widehat{y}}_{i,t}={\mu }_{i}+{y}_{i,t-1}+{\varphi }_{1}\left({y}_{i,t-1}\right).$$

A large number of other combinations of the parameters $p$, $d$ and $q$ is also possible. We used the “forecast” package in R^24^ to simulate the combination resulting in the lowest AIC value given the input series via the auto.arima() function. This function estimates the parameters of the AR and MA processes using Maximum Likelihood Estimation (MLE).

#### Multivariate spatio-temporal predictions using INLA

The spatio-temporal model includes a component to account for temporal autocorrelation as well as a component to account for spatial autocorrelation between neighboring areas. We consider the following model:

7 $${\widehat{y}}_{i,t}={\widehat{\beta }}_{0}+{\sum }_{k=1}^{K}{\widehat{\beta }}_{k}{x}_{k,i,t-1}+{\sum }_{h=1}^{H}{f}_{h}\left({z}_{h,i,t-1}\right)+{\varepsilon }_{i,t},$$

where ${\widehat{y}}_{i,t}$ is the predicted positivity in area $i$ for week $t$. The model consists of intercept ${\widehat{\beta }}_{0}$, some linear covariates ${\varvec{x}}=\left({x}_{1},\dots ,{x}_{K}\right)$ multiplied by their respective coefficients ${\widehat{\beta }}_{k}$, and a set of functions ${\varvec{f}}=\left\{{f}_{1}(\cdot ),\dots ,{f}_{H}(\cdot )\right\}$ defined in terms of its covariates ${\varvec{z}}=\left({z}_{1},\dots ,{z}_{H}\right)$. The form of the functions ${f}_{h}(\cdot )$ can be varied to adjust for the spatial and temporal autocorrelation in the model. Finally, ${\varepsilon }_{i,t}$ is the error which is assumed ${\varepsilon }_{i,t}\sim Normal\left(0,{\sigma }^{2}\right)$.

In this model, we consider the following covariates based on their significance in a majority of the training weeks: average positivity of areas neighboring area $i$ in the previous week $t-1$, number of tests in area $i$ per 100,000 adults in the previous week $t-1$, the number of emergency calls assessed as suspected COVID-19 by ambulance personnel per 100,000 adult inhabitants in area $i$ in the previous week $t-1$, population density in area $i$, week $t-1$ average of the daily percentage change in visitors to and from workspaces compared to a baseline day in area $i$, and a binary variable indicating whether area $i$ was in the top 10 of highest positivity in the previous week $t-1$. A covariate was considered different from zero when the 95% credible interval of the posterior distribution of the estimates did not contain zero.

Besides these linear covariates, we include a function for the autoregressive structure in the time series, with an order of 1. We modelled the spatial dependencies between neighboring areas using the Besag–York–Mollie (BYM) specification^25^. Following, we define ${\upsilon }_{i}={f}_{1}(i)$ as the area specific effect, where the spatially structured residual ${\upsilon }_{i}$ is modelled using an intrinsic conditional autoregressive structure (iCAR):

$$\upsilon_{i} \left| {\upsilon_{j \ne i} \sim Normal\left( {m_{i} ,\,S_{i}^{2} } \right)} \right.,$$

$$m_{i} \, = \,\frac{{\sum {_{{j \in N\left( i \right) }} }\upsilon_{j} }}{\# N\left( i \right)}\,{\text{and}}\,S_{i}^{2} \, = \,\frac{{\sigma_{\upsilon }^{2} }}{\# N\left( i \right)},$$

where $\#N(i)$ indicates the number of neighboring areas sharing boundaries with area $i$. Finally, the model includes an unstructured spatio-temporal interaction component.

We used the ‘INLA’ package in R for Bayesian estimation and interference of all model parameters^26^. Compared to more traditional Bayesian approaches using Markov Chain Monte Carlo (MCMC) simulations, INLA provides estimates in a computation time which is considerably shorter, while the approximation is as good or even better than the estimates provided by MCMC^27^. We used uninformative priors for all parameters.

#### Ensemble model

At the end of the modelling period, we evaluated whether a weighted ensemble of the predictions of the four models would have improved the results. The observed positivity ${y}_{i,t}$ is equal to a weighted average of the different models $v$ in $(RF, GB, INLA, ARIMA)$:

8 $${y}_{i,t}={\omega }_{0}+{\sum }_{v}{\omega }_{v}{\widehat{y}}_{i,t,v},$$

where ${\omega }_{0}$ is the intercept and ${\omega }_{v}$ is the weight of model $v$. The weights were estimated using ordinary least squares estimation. The naïve model was excluded as it is the baseline model to which all other models are compared, including the ensemble model.

### Ethical declaration

All parts of the study were conducted in accordance with the Declaration of Helsinki by the World Medical Association, as revised in 2013. The study was approved by the Ethical Review Board in Sweden (Etikprövningsmyndigheten, application 2020-04210 and 2021-01915), who waived the need for informed consent since only information on aggregate group level was extracted.

## Results

### Gradient boosting

Parameter tuning was reiterated on each time step of the cumulative calculations, because the estimated optimal parameters changed when more and more weekly data were added to the training set. The four parameters tuned to optimize GB varied between the values shown in Table S2 in the Supplementary Materials. The parameters stayed well within the chosen tuning ranges, indicating enough freedom for the model to optimize, with the exception of *n.trees* which was forced not to go below 300. The optimal shrinkage was always 0.2 except for the very first training set. Therefore, fixing it to 0.2 could substantially save computer runtime by reducing the computational cost of optimizing again for every training set. It was observed in a number of test runs that the temporal evolution of the RMSE changed only marginally when the range or the resolution of the tuning grid was changed, indicating that the model was robust to changes in the parameters.

**Figure 1:**
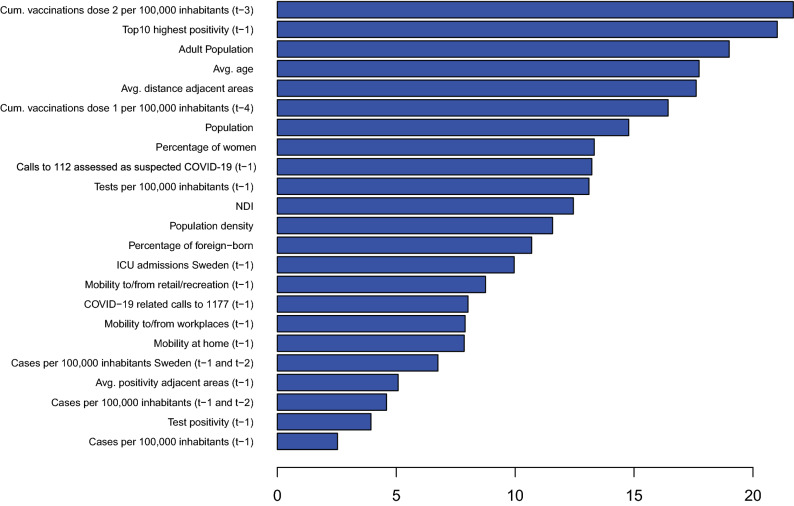
Gradient boosting, averaged ranking (over time) of importance for each variable. Lower ranks indicate higher importance.

The variable importance based on the Gini index was recorded on each time step of the cumulative calculations. It changed remarkably between different time points. Figure 1 shows the variable importance averaged over the entire time series, where a lower rank indicates higher importance. It shows that cases per 100,000 inhabitants (week $t-1$), cases per 100,000 inhabitants (week $t-1$ and week $t-2$ combined), lagged test positivity (week $t-1$), and average positivity of adjacent areas (week $t-1$) played the most important role, the third pointing at temporal autocorrelation and the latter pointing at some spatial autocorrelation.

The RMSE obtained by comparison of the predicted positivity and the true rates (Eq. (1)) was compared to the mean RMSE calculated during the cross validation, as shown in Fig. S1 in the Supplementary Materials. The estimated mean RMSE—based on the training data—is mostly lower and develops much smoother. This can be a reference to possible overfitting, especially when the values of the variables change sharply each week.

### Random forest

The only parameter tuned for RF was the number of variables sampled to make the splits in the decision trees (*mtry*). The number of trees constructed for each forest (*ntree*) was held constant at 1000, which is well above the default (*ntree* = 500), therewith ensuring convergence. It turned out that the optimal *mtry* value varied between 1 and 6 during the cumulative calculations, while the tuning grid range was 1 to 15.

**Figure 2:**
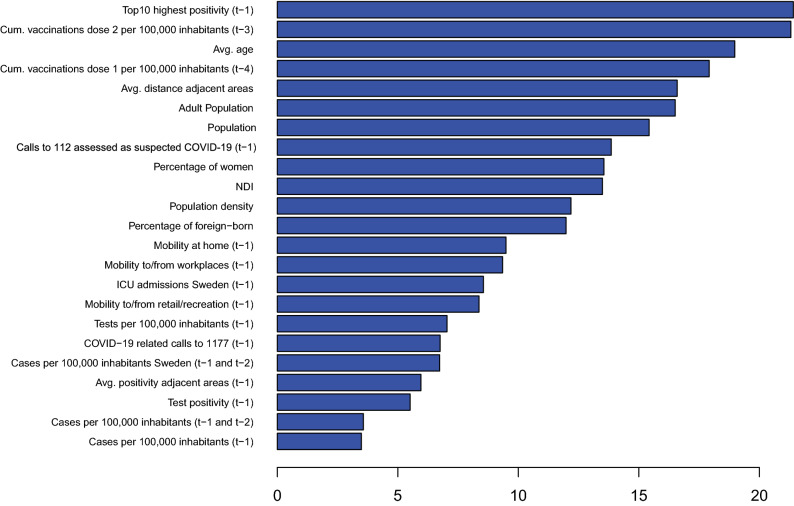
Means of the importance ranks over all time points for the Random Forest model. Lower ranks indicate higher importance.

The means of the ranks over all time points (Fig. 2) are very similar to those obtained for GB: cases per 100,000 inhabitants (week $t-1$), cases per 100,000 inhabitants (week $t-1$ and week $t-2$ combined), lagged test positivity (week $t-1$), and average positivity of adjacent areas (week $t-1$) are the most important predictors, pointing to the existence of some spatial and temporal correlation.

Also for the RF model, overfitting seems to play a role: the mean RMSE estimated by cross validation during training is mostly lower than the real one calculated by Eq. (1), as shown in Fig. S2 in the Supplementary Materials. Moreover, the curve based on the estimated mean RMSE is much smoother. The deviations are especially big when the incidence changes sharply.

### Autoregressive integrated moving average (ARIMA)

The ARIMA model trained and predicted ${\widehat{y}}_{i,t}$ for each area $i$ independently, resulting in 50 separate models. Table S3 in the Supplementary Materials shows the distribution of different sets of parameters over the different areas. The time series in most areas (32%) followed the structure of no history dependencies, ARIMA (0, 0, 0). In fifteen areas (30%), the time series followed the structure of a first-order autoregressive model with one order of non-seasonal differencing and a constant term. The remainder of the areas had varying combinations of parameters $p$, $q$ and $r$. However, the time series for all areas have at least one differentiation, which indicates that none of the time series are stationary in level. Nine areas (18%) have at least one MA parameter, which shows that the time series correct themselves over time against a long-term average. Based on the results in Table S3, positivity rate is clearly dominated by a random walk process with a large element of fluctuations over time that cannot be explained by historical values of positivity rate alone.

### Integrated nested Laplace approximations (INLA)

**Figure 3:**
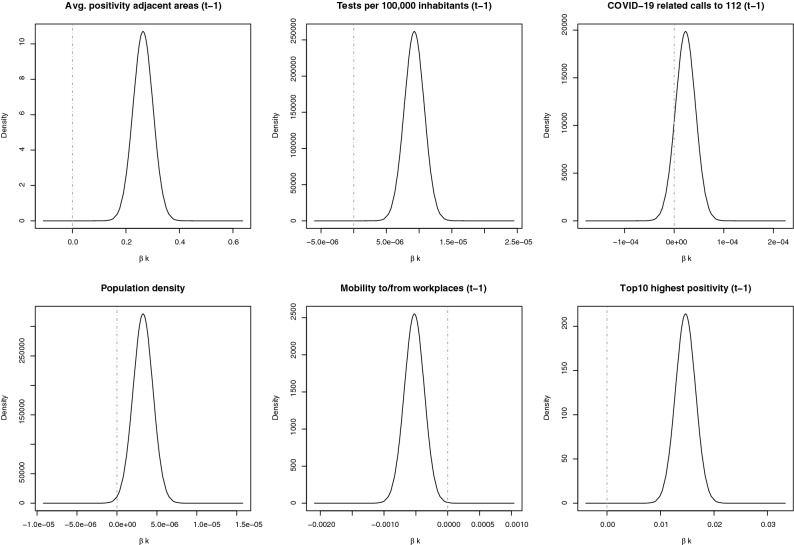
Posterior distributions for ${\upbeta }_{\mathrm{k}}$ in prediction model for week 21, 2021. The gray dot-dashed lines indicate zero.

The INLA provided Bayesian estimates for the fixed parameters (intercept and linear covariates) as well as the random effects (spatial autocorrelation, temporal autocorrelation and spatio-temporal interaction effects). Rather than a single estimate for the coefficients in the model, INLA provides a posterior distribution of the parameters, which allows for evaluation of its uncertainty and significance. Since the model is retrained every week as new data comes in, the posterior distributions of the parameters also vary on a weekly basis. Figure 3 shows the posterior distributions for ${\beta }_{k}$ of the six covariates included in the model, for week 21, 2021. A parameter is considered significant when zero is outside the 95% credible interval. Please note that no variables were scaled, so depending on the units, ${\beta }_{\mathrm{k}}$ can take very small numbers despite being significant. The intercept ${\beta }_{0}$ was not significantly different from zero.

As can be derived from the posterior distributions, average positivity in the neighboring areas had a large effect on positivity in the next week. Similarly, large positive relations are observed with the number of tests per 100,000 inhabitants and the binary variable indicating whether the area was in the top 10 highest positivity last week. Small but significant relations were also observed with population density. The significance of the relation with the number of calls to the emergency line 112 assessed as suspected COVID-19 was varying over time. In the example of week 21, 2021 shown in Fig. 3, the calls to 112 had no significant impact, but it was included in the model since it was significant for a large part of the other weeks. A negative relation was found between predicted positivity and average of the daily percentage change in visitors to and from workspaces. This is the opposite of the expected, as one would expect positivity to increase with increasing travels to workplaces, but this inverse relation is likely caused by restrictions that were applied, e.g. more people were asked to work from home when the severity of the pandemic and the positivity rates increased. A lag effect between restrictions and their effect on positivity rates might have caused this inverse relationship.

The covariate accounting for average positivity in the neighboring areas took care of part of the spatial autocorrelation structure in the data. Similarly, the number of tests per 100,000 inhabitants, the number of calls to the emergency line 112 assessed as suspected COVID-19, and previous week’s top 10 code, all accounted for part of the temporal correlation in the data as well as part of the spatio-temporal interaction effects. Therefore, the coefficients for the random effects accounting for remaining spatial, temporal and spatio-temporal autocorrelation were small and mostly not significantly different from zero, with a few exceptions depending on week $t$ and area $i$.

### Model comparison

**Figure 4:**
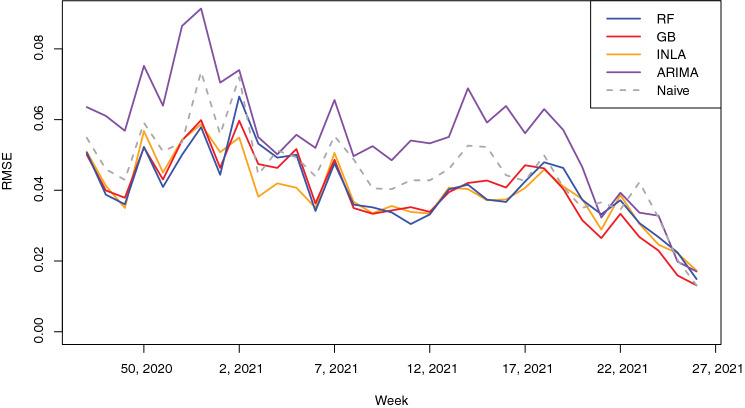
RMSE for the different models over time.

Performance of all models was compared to each other and to the naïve model based on the RMSE values. Since the RMSE value is computed each week as new data comes in, we can compare model performance over time. Figure 4 shows the progress of the model performance over time. Please note that the first 20 weeks were used as training data before the first RMSE was computed. Therefore, the time series does not span the complete year, but includes both the second and third wave of the pandemic. These waves are also visible in the RMSE time series, indicating a decrease in model performance when positivity values increased. The figure also clearly shows that the models improved performance after week 2, 2021, marking the end of the second wave of the pandemic (i.e. a strong increase and decrease in the response variable), which was the first wave seen by the model. The RF, GB and INLA models significantly (p-value < 0.001) outperformed the naïve model and ARIMA model, but their performance was not significantly different from each other.

**Figure 5:**
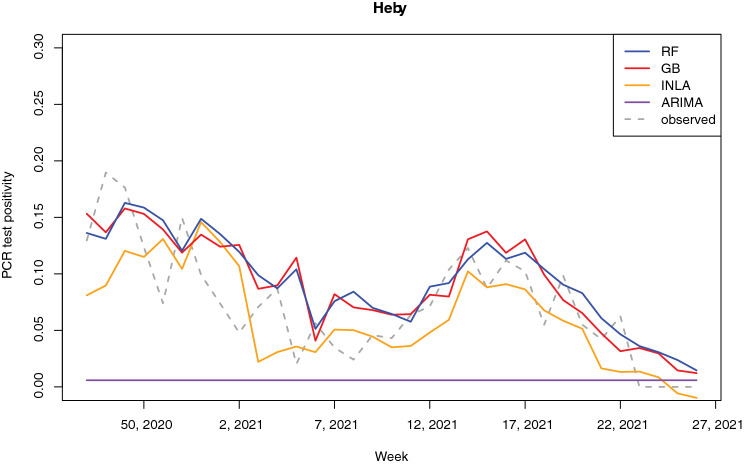
Time series of predicted and observed test positivity in Heby, a part of the region less strongly affected by the pandemic.

**Figure 6:**
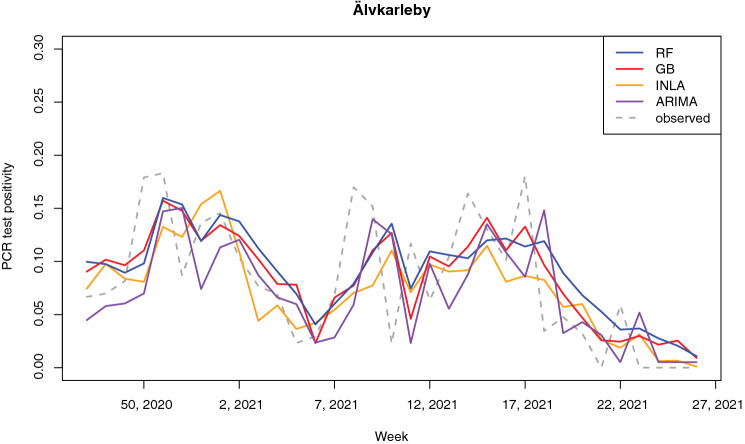
Time series of observed and predicted test positivity in Älvkarleby, a part of the region strongly affected by the pandemic.

Figures 5 and 6 show the time series of the predicted positivity in the areas of Heby and Älvkarleby, respectively. These areas show examples of an area where the number of cases remained relatively low throughout the pandemic (Heby) and an area more strongly affected by the pandemic (Älvkarleby). The random forest model, gradient boosting model and INLA model are able to capture the two infection waves well (note that these are the second and third infection wave, as no data was available at the time of the first wave). The ARIMA model was not able to capture any temporal variability for Heby, due to a combination of parameters $p,d,q$ of (0, 0, 0). This combination occurred in 8 out of the 50 areas (Table S3 in the Supplementary Materials). In both Heby and Älvkarleby, the predictions lag the observed results.

**Figure 7:**
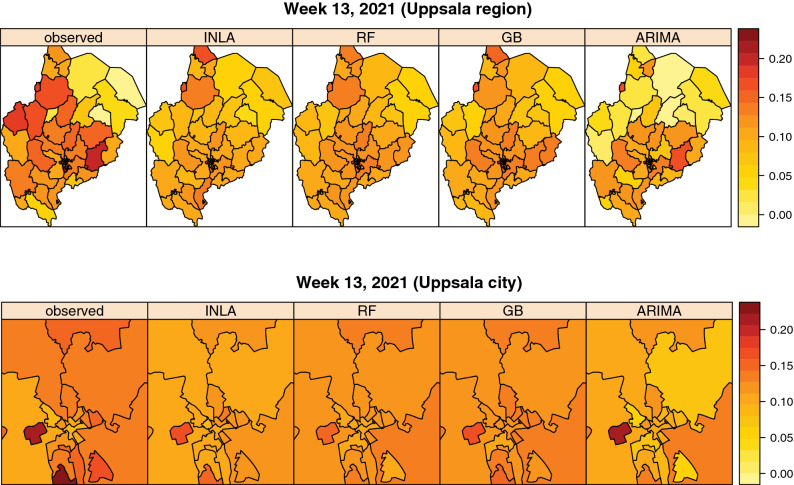
Maps of observed and predicted test positivity in week 13, 2021, at the peak of the third wave of the pandemic. Uppsala county (top) and zoom-in of Uppsala city (bottom).

Figure 7 shows maps of the observed and predicted positivity in week 13, 2021, at the peak of the third infection wave of the pandemic. The INLA, RF and GB models capture some spatial variability through their spatial covariates, but none of the models is able to capture the same amount of spatial variability as found in the observed positivity. Especially within the city (bottom of Fig. 7), we observe that INLA, RF and GB capture the spatial variability quite well. In the rural parts of Uppsala County (top part of Fig. 7) the spatial variability is captured less consistently.

Finally, we evaluated whether a linear weighted ensemble of the predictions of the four models would improve the results. The estimated weights were ${\omega }_{INLA}=0.4, { \omega }_{RF}=0.4$ and ${\omega }_{GB}=0.2$. The model included no intercept (${\omega }_{0}=0$). The ARIMA model was excluded since its weight ${\omega }_{ARIMA}$ was estimated to be zero and did not significantly improve model performance. The weighted ensemble slightly improved the RMSE to a mean of 0.039 over the entire time series, versus 0.040 for the RF, GB and INLA models, 0.055 for the ARIMA model and 0.046 for the naïve model. The weighted ensemble outperformed all other models based on the paired Wilcoxon signed-rank test (p-value 0.02 for ensemble vs. GB, p-values < 0.001 for ensemble vs. the other models).

Paired Wilcoxon signed-rank tests were used to compare the predictive performance of the different models and test their significant difference. Since the independence between samples could not be guaranteed given the temporal autocorrelation in the RMSE time series, we have run additional tests to evaluate whether the differences between the RMSE time series were significantly different from zero ($\alpha =0.05$) after accounting for a potential AR1 process in the data. These tests yielded the same conclusions as the paired Wilcoxon signed-rank tests.

## Discussion and conclusions

Three out of four models outperformed the naïve model in predicting test positivity at a local level in Uppsala County, Sweden, and demonstrated a moderate accuracy. The RF and GB models showed similar performance, which was expected as these methods are both based on decision trees and used the same set of covariates. Their similarity in predictive capabilities became particularly clear when examining the time course of the RMSE and at the averaged ranks. Interestingly, the INLA model also showed similar performance to RF and GB models throughout the entire year, despite being dependent on a completely different model and a different selection of covariates. For example, the GB and RF models relied heavily on cases per 100,000 inhabitants and 1177 call data, whereas the INLA model relied on 112 emergency call data and top 10 code, indicating whether an area was in the top 10 of highest positivity in the previous week. For all models, lagged positivity and positivity in the neighboring areas were important covariates, and Google Mobility data was relatively important. An ensemble model combining the weighted predictions of GB, RF and INLA slightly improved the predictive performance. It should be noted, however, that the ensemble model we used is of simple nature, in an effort to see if a linear combination of the different model predictions would lead to any improvement in the model predictions. A suggestion for future research would be to explore different and more complex ensemble models available in machine learning literature.

A fine geographical resolution is essential to guide local testing strategies, and test positivity has been suggested as one of the main criteria to be considered for the assessment of epidemic control^12^. For example, a high test positivity can be an indication of increased community transmission and delayed case identification. Increased percentages of positive samples can also indicate that the testing guidelines and strategies are mainly targeting symptomatic patients and that the testing capacity is not wide enough to include all possible exposed contacts and COVID-19 asymptomatic patients that would require isolation and further contact tracing. High test positivity rates could as well be the result of insufficient local testing supplies or restricted access to testing facilities. On that account, Uppsala County Council did make use of two mobile testing stations during 2020 and 2021, that were strategically relocated to target areas manifesting the highest positivity rates during the current week (current positivity). We here show that using a prediction model as ours has the potential to improve the identification of areas at high risk of exhibiting high positivity the following week compared to relying on current positivity only. With our weekly prediction model, we show that the model can improve the identification of areas at high risk of exhibiting high positivity the following week compared to relying on current positivity only.

A strength in our study is that we have used four different models in the same environment, in a geographically well-defined area with access to comprehensive health care data, enabling a multipoint view of the status of the pandemic that may be impacted in an asynchronous manner by the spread of the infection over time. Our dataset was updated weekly, our models were constantly calibrated and the parameters were refined based on the evolving scientific and empirical knowledge regarding infection and transmission rates. By incorporating new knowledge and data each week from multiple resources, our models were gradually based on more and more data, which is reflected in an increase in accuracy over time.

The temporal predictive performance of the RF, GB and INLA models was reasonably good. Despite the inability to capture strong peaks and sudden changes in positivity, these three models were all able to capture major trends and infection waves. The lagged test positivity was one of the most important predictors for RF, INLA and GB. This might be part of the explanation of the apparent lag in predictions observed in Figs. 5 and 6. However, these models all still outperformed the naïve model, which is purely based on a one-week lagged test positivity, indicating that the other covariates contributed to predictive performance but that more data would be needed for more accurate prediction. The performance of ARIMA differed largely between areas, which all had individual models with individual sets of parameters. A large part of the models depended on predicting similar values to the week before, identical to the naïve model used for comparison. For some areas, predictions were worse (e.g. predicting a constant value as seen e.g. in Fig. 5) whereas for other areas predictions were more sophisticated, showing ability to model long-term temporal trends in the data. Our models provided short-term predictions of one week ahead, in line with the intended purpose to relocate mobile testing stations where they were needed the most. It is likely that the models lose predictive power when aiming to predict longer periods of time, due to large temporal variations in public restrictions in mobility and social contact, as well as the emergence of new variants and other seasonal factors like weather conditions.

The GB and RF models included area-specific covariates which indirectly captured some spatial variability (e.g. socio-economic neighborhood characteristics and positivity of neighboring areas). The intention of the INLA model was to capture spatial variability both through these spatial covariates as well as through the spatial autocorrelation structure in the data. However, the model did not capture more spatial variability than what was covered by the spatial covariates. This was however not surprising, as one of the covariates included positivity of neighboring areas, which covers the same spatial structure as also covered by the spatial autocorrelation model. The GB, RF and INLA models all captured the spatial variability within Uppsala City better than in the rural areas of the region. This is likely due to less interaction between the rural areas than between the smaller service point areas within the city. Besides that, the rural areas on the outskirts of the region have more interaction with cities in neighboring counties rather than Uppsala City, while data from neighboring counties was not included. The ARIMA model was completely based on temporal autocorrelation and was therefore not designed to capture any spatial variability.

With regards to the spatial units, it was important for us to be able to make predictions at the finest level possible. We wanted to bring into light vulnerable neighborhoods and to understand which areas were in most need of targeted testing efforts, so that prevention efforts could be tailored to the local needs. However, we did not use postal code level resolution, because the population size in many units was too small and leading to unreliable predictions. City parts defined by the municipality were also inadequate since their wide areal range did not reveal local outbreaks at an early stage. Instead, we decided to use a service point area as resolution unit, thus incorporating probable exposure to the virus while visiting adjacent supermarkets, pharmacies and using public transport. In Sweden, commercial building complexes accommodate large grocery and retail stores, recreational areas, as well as unique national postal service points where local residents from neighboring postal codes with similar demographic and socioeconomic characteristics gather in big numbers. Those urban cross points represented by the postal service point area is where a substantial spread of COVID-19 may be taking place.

A limitation that may have compromised our ability to predict the test positivity was the absence of detailed data on individual mobility in daily activity patterns between the different areas and from neighboring counties. Furthermore, our models did not account for the effect of major public events or the varying severity over time of restriction measures imposed by the government. Another potential limitation was the lower testing rates observed in Sweden in neighbourhoods characterized by socioeconomic deprivation^28^, which may entail differences in case notification rates and test positivity as compared to more affluent areas. However, the inclusion of ‘NDI’ and ‘proportion of inhabitants with foreign background’ across postal code areas in our models yielded only moderate importance in RF and GB, and did not influence the INLA model. Furthermore, Uppsala County Council could only provide either assisted on-site testing that required a pre-booked appointment or drop-in testing at designated locations during certain time periods. At-home testing options could have increased the testing rates in areas far from testing stations, while maintaining a central reporting system of self-test results would have been beneficial. Meanwhile, sewage analysis of SARS-CoV-2 has been shown as a valid tool for measuring the epidemic^29^, however, in Uppsala County, only restricted local data was available and only for limited time periods, and on the country-side level, some areas are not linked to the municipality sewage system. A suggestion for future epidemics is to collect data from different sources and scale levels in a systematic manner to be able to better capture trends and aid in prediction efforts. Finally, we also experienced some delays and/or revisions in the data, that frequently caused an underestimation of the number of hospitalized and vaccinated inhabitants in the areas at the moment when the predictions were made, even though this discrepancy is retrospectively rectified within a matter of weeks. As an example, patients in the hospital could have their diagnoses updated as further tests results came in, retrospectively re-classifying them into COVID-19 patients. Nevertheless, even if revisions are repeatedly required, when the overall quality of the data is good and consistent, the existing indicators can still offer a good understanding of the transmission trends over time and in space^5^ as long as there is a good understanding of the limitations and the direction of the potential inaccuracies.

There have been numerous attempts to accurately forecast COVID-19 with a variety of methods, indicators and a wide range of precision and accuracy^11^. Da Silva et al.^9^ found that linear regression and artificial neural networks (ANN) gave the best performance compared to support vector regression (SVR) and RF when attempting to spatially predict COVID-19 cases and deaths in Brazil. They also suspected that these methods might have had an advantage when applied to the Brazilian context considering that the spread of infection recorded a linear trajectory during the study period. Other studies predicting COVID-19 cases by employing ARIMA in Ethiopia^30^ and Vector Autoregression in the United States^10^ yielded good accuracy, while long short-term memory (LSTM) and the bidirectional LSTM were found to be more robust and accurate than ARIMA and SVR using data from China^31^. In our study, we find a rather similar performance using different methods, indicating that the quality and types of input data might be the limiting step in improving predictions. In general, transmission of viruses at any given setting is dependent on the surrounding environmental conditions that can largely differ from country to country. Such conditions are either controlled by humans (government policies, restriction in movement, possibility to work from home, density of public transportation networks) or they simply cannot be anticipated (biological properties of the virus, climate features). It is therefore not realistic to expect that one single prediction model on the transmission of a virus can be a global standard, rather a wide variety of models to choose from depending on the circumstances of each country and available data^11^. Our study identified some important predictors for test positivity which are worth investigating in different countries and future models when similar data is available. In particular, calls to the nurse’s help line and to the emergency hotline were variables that were informative to the model and that should be possible to access also in other countries.

Local prediction models are likely not generalizable across countries and continents, among other reasons due to variations in the availability of data, differences in the timing and intensity of restrictions in mobility and social contact, the presence of distinct cross-cultural characteristics in social interactions and networks as well as differences in local climate and weather However, by understanding the inevitable limitations that accompany such prediction models, we can appreciate that various types of data can be informative and that local fitting of data is necessary. Combining various sources of data into prediction models can aid in local efforts to curb the spread of viral disease.

## Supplementary Information

Supplementary Information.

## Data Availability

The data used in this study is available online at: https://github.com/MolEpicUU/spatiotemporal_predictions_COVID19.
