# Supplementary material for: Spatio-temporal predictions of COVID-19 test positivity in Uppsala County, Sweden: a comparative approach
Source: Sci Rep. 2022 Sep 7;12:15176. doi: 10.1038/s41598-022-19155-y (PMC9450842; doi:10.1038/s41598-022-19155-y)
Supplement: Supplementary file 1 — Supplementary Information. [file 41598_2022_19155_MOESM1_ESM.pdf]

Supplementary materials to

"Spatio-temporal predictions of COVID-19 test positivity in Uppsala County, Sweden – a comparative approach"

Vera van Zoest [1], Georgios Varotsis[2], Uwe Menzel[2], Anders Wigren[2], Beatrice Kennedy[2], Mats Martinell[3,4], Tove Fall[2]

[1] Uppsala University, Department of Information Technology, Division of Computer Systems, P.O. Box 337, SE-751 05 Uppsala, Sweden

[2] Department of Medical Sciences, Molecular Epidemiology and Science for Life Laboratory, Uppsala University, 751 85 Uppsala, Sweden

[3] Department of Public Health and Caring Sciences, Uppsala University, 751 22 Uppsala, Sweden

[4] Primary Care and Health, Region Uppsala, 753 31 Uppsala, Sweden

Table S1. Name, description and type of all the variables that were used in the different prediction models

Variable	Description	Data type
**Service Point**	Name of area i corresponding to a unique PostNord service point.	Fixed nominal
**Postal code**	The postal code corresponding to the service point of area i.	Fixed nominal
**Municipality**	The name of the municipality corresponding to area i.	Fixed nominal
**Week**	Week number of the week $t-1$.	Time-dependent ordinal
	**Spatial features**	
**Location of service point**	The coordinates of each area i within Uppsala County were collected from SCB [1]. The libraries rgdal and sp were used in R to transform the coordinate system of each area i to WGS84.	Fixed continuous numerical
**Average distance (km) from adjacent areas**	Average distance in kilometers of each area i from the surrounding areas within Uppsala County with which they share at least one contact point between their administrative borders within Uppsala County. The geodesic distances were measured using the R package 'geodist' that applies the methods suggested in [2].	Fixed continuous numerical
	**Demographic characteristics (all from Statistics Sweden, access date 2019-12-31)**	
**Population**	Total population of area i on December 31, 2019.	Fixed (for the duration of the study) continuous numerical
**Adult population**	Population including individuals 16 years of age and older of area i on December 31, 2019.	Fixed (for the duration of the study) continuous numerical
**Population density**	Number of people (total population) residing within the administrative borders of area i per square kilometer of area i on December 31, 2019.	Fixed (for the duration of the study) continuous numerical
**Average age**	Average age of the population found within the administrative borders of area i administrative borders.	Fixed (for the duration of the study) continuous numerical
**Percentage of foreign-born**	Percentage of the population residing within the administrative borders of area i that were born foreign outside Sweden.	Fixed (for the duration of the study) continuous numerical
**Percentage of women**	Percentage of the population residing within the administrative borders of area i that are registered as female.	Fixed (for the duration of the study) continuous numerical
**Neighborhood Deprivation Index (NDI)**	The NDI of the population residing within the administrative borders of area i was calculated using a principal component analysis based on the yearly median net income of each area i and the proportion of employees, students and individuals	Fixed (for the duration of the study) continuous numerical

	holding an academic degree among the adult population of each area i [3]. The deprivation index was generated after dividing the first principal component by the square root of its eigenvalue. With this method, an area with overall unfavorable socioeconomic circumstances receives a low NDI value.	
**Direct indicators of SARS-CoV-2 spread (data from Uppsala County Council)**		
**Test positivity (week $t-1$)**	The percentage of all COVID-19 RT-PCR tests that were positive per area i during week $t-1$.	Time-dependent continuous numerical
**Test positivity (week t)**	The percentage of all COVID-19 RT-PCR tests that were positive per area i during week t. This is the outcome variable.	Time-dependent continuous numerical
**Cases per 100,000 inhabitants (week $t-1$)**	The total number of positive COVID-19 RT-PCR tests per 100,000 inhabitants of area i (adult population) and during week $t-1$.	Time-dependent continuous numerical
**Cases per 100,000 inhabitants (during weeks $t-1$ and $t-2$)**	The total number of positive COVID-19 RT-PCR tests per 100,000 inhabitants of area i (adult population) during weeks $t-1$ and $t-2$.	Time-dependent continuous numerical
**Tests per 100,000 inhabitants (week $t-1$)**	The total number of COVID-19 RT-PCR tests performed per 100,000 inhabitants (adult population) and week $t-1$.	Time-dependent continuous numerical
**Average positivity of adjacent areas (week $t-1$)**	Average test positivity per week $t-1$ of the areas within Uppsala County that share at least one contact point between their administrative borders and area i.	Time-dependent continuous numerical
**Top 10 with highest test positivity (week $t-1$)**	Whether the area i was among the top 10 areas with the highest test positivity during week $t-1$. Yes=1; No=0.	Time-dependent binary nominal
**ECDC risk level (week $t-1$)**	Area i marked in the colors ‘dark red’, ’red’, ’orange’, ’green’ or ’grey‘ based on the risk levels suggested by the Council Recommendation on a coordinated approach to travel measures in the EU (amendment of January 28, 2021 of the European Centre for Disease Prevention and Control (ECDC) [4].	Time-dependent ordinal
**Hospital and ICU beds occupied by patients from each area (data from Uppsala County Council)**		
**Average number of patients (week $t-1$)**	A 7-day average of the number of occupied hospital beds during week $t-1$ in the municipality corresponding to area i.	Time-dependent continuous numerical
**Cases per 100,000 inhabitants in Sweden (during weeks $t-1$ and $t-2$)**	The total number of positive COVID-19 RT-PCR tests in the entire country (Sweden) per 100,000 inhabitants (total population) during weeks $t-1$ and $t-2$ [5].	Time-dependent continuous numerical
**Admissions in the Intensive Care Units (ICU) in Sweden**	Number of admissions in the Intensive Care Units (ICU) in the entire country (Sweden) during $t-1$ and $t-2$ [5].	Time-dependent continuous numerical

**(during weeks $t-1$ and $t-2$)**		
**Indirect indicators**		
**COVID-19-related calls to 1177 per 100,000 inhabitants (week $t-1$)**	Number of calls with COVID-19-related symptoms to the 1177 Healthcare Advice Line per 100,000 inhabitants (adult population) in area i and week $t-1$.	Time-dependent continuous numerical
**Number of calls assessed as suspected COVID-19 by the ambulance personnel per 100,000 inhabitants (week $t-1$)**	Number of ambulance calls to the 112 emergency line per 100,000 inhabitants (adult population) in area i and week $t-1$ which were assessed as suspected COVID-19 by the ambulance personnel.	Time-dependent continuous numerical
**Vaccination coverage**		
**Total vaccinations (dose 1) per 100,000 inhabitants at least 3 weeks ago**	Cumulative number of vaccinated individuals per 100,000 inhabitants (total population) residing within the administrative borders of area i who have received the first dose of an EMA-approved vaccine against COVID-19 at least three weeks before week t.	Time-dependent continuous numerical
**Total vaccinations (dose 2) per 100,000 inhabitants at least 2 weeks ago**	Cumulative number of vaccinated individuals per 100,000 inhabitants (total population) residing within the administrative borders of area i who have received the second dose of an EMA-approved vaccine against COVID-19 at least two weeks before week t.	Time-dependent continuous numerical
**Indicators of increased social mobility from Google Mobility**		
**Mobility to and from workplaces (week $t-1$)**	Weekly average of the daily percentage change in visitors to and from workspaces during week $t-1$ compared to a baseline day for the municipality corresponding to the area i [6]	Time-dependent continuous numerical
**Mobility to and from retail and recreation (week $t-1$)**	Weekly average of the daily percentage change in visitors to and from retail stores and recreational activities during week $t-1$ compared to a baseline day for the municipality corresponding to the area i [6]	Time-dependent continuous numerical
**Mobility at home (week $t-1$)**	Weekly average of the daily % change in duration spent at home during week $t-1$ compared to a baseline day for the municipality corresponding to the area i [6]	Time-dependent continuous numerical

Table S2. Optimal model parameter ranges estimated during tuning.

**Description**	**Variable**	**Min**	**Max**	**Default**
Total number of trees	*n.trees*	300	900	500
Learning rate (step-size reduction)	*shrinkage*	0.001	0.2	0.1
Minimum number of samples in terminal nodes	*n.minobsinnode*	10	30	10
Maximum depth of trees	*interaction.depth*	1	4	1

Table S3. Distribution of the parameters used in the ARIMA models for different areas i.

Parameters p, d, q	Count
0,1,0	16
0,0,0	8
1,1,0	6
1,0,0	4
1,2,0	3
0,1,1	2
0,2,1	2
0,0,1	1
0,2,0	1
0,2,2	1
1,0,1	1
1,1,1	1
2,0,0	1
2,0,1	1
2,1,0	1
2,2,0	1

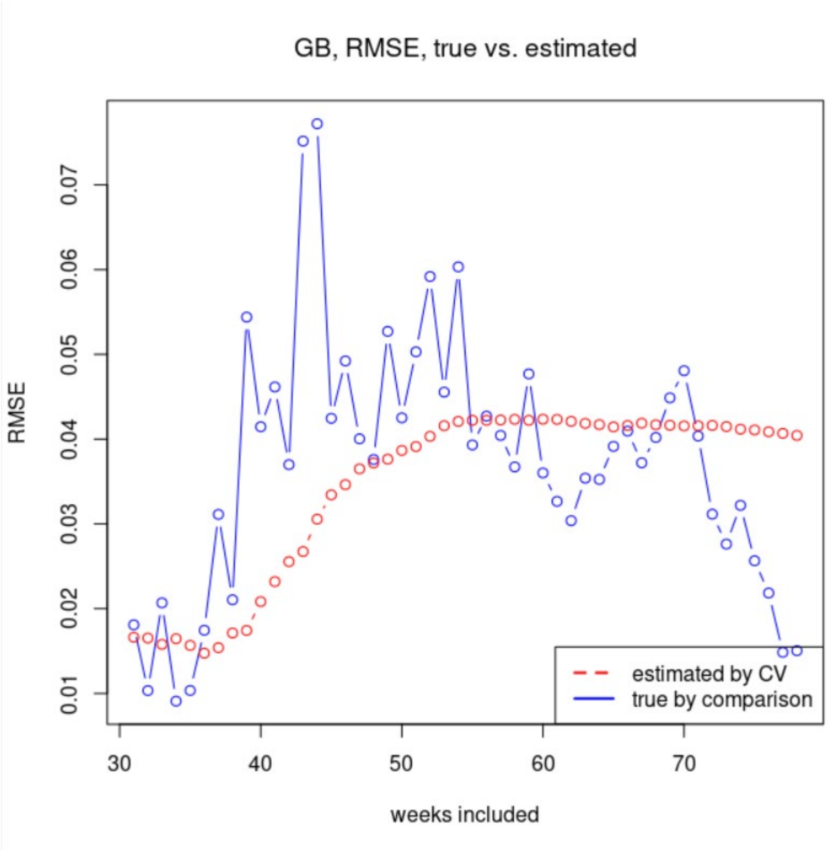

Figure S1. Gradient Boosting: the RMSE obtained by comparison with the predicted positivity and the true rates and the mean RMSE calculated during the cross validation (CV).

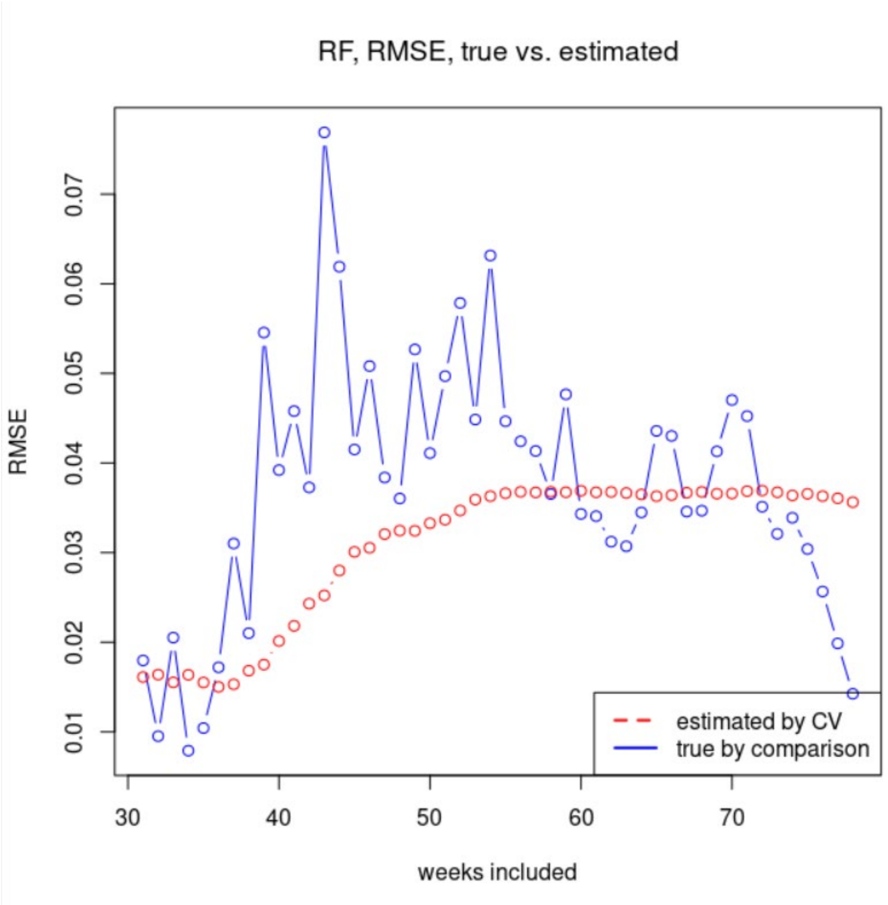

Figure S2. Random Forest: the RMSE obtained by comparison with the predicted positivity and the true rates and the mean RMSE calculated during the cross validation (CV).

References

1 SCB. *Statistical database - Environment, Statistics Sweden (SCB)*, <https://www.statistikdatabasen.scb.se/pxweb/en/ssd/START__MI/> (2020).

2 Karney, C. F. F. Algorithms for geodesics. Journal of Geodesy 87, 43-55, doi:10.1007/s00190-012-0578-z (2013).

3 Messer, L. C. et al. The development of a standardized neighborhood deprivation index. J Urban Health 83, 1041-1062, doi:10.1007/s11524-006-9094-x (2006).

4 ECDC. "Maps in support of the Council Recommendation on a coordinated approach to travel measures in the EU." https://www.consilium.europa.eu/media/48122/st05716-en21-public.pdf (2021).

5 Fohm, F. "Bekräftade fall i Sverige – daglig uppdatering." https://www.folkhalsomyndigheten.se/smittskydd-beredskap/utbrott/aktuella-utbrott/covid-19/statistik-och-analyser/bekraftade-fall-i-sverige/ (2021).

6 Google LCC. "Google COVID-19 Community Mobility Reports." Retrieved July 8, 2021, from https://www.google.com/covid19/mobility/ (2021).
